# Prevalence of and Eligibility for Surveillance Without Anticoagulation Among Adults With Lower-Risk Acute Subsegmental Pulmonary Embolism

**DOI:** 10.1001/jamanetworkopen.2023.26898

**Published:** 2023-08-02

**Authors:** Samuel G. Rouleau, Mahesh J. Balasubramanian, Jie Huang, Tad Antognini, Mary E. Reed, David R. Vinson

**Affiliations:** 1 Department of Emergency Medicine, UC Davis Health, University of California, Davis, Sacramento; 2 The Permanente Medical Group, Oakland, California; 3 Department of Adult Hospital Medicine, Kaiser Permanente Roseville Medical Center, Roseville, California; 4 Division of Research, Kaiser Permanente Northern California, Oakland; 5 Department of Adult and Family Medicine, Kaiser Permanente Santa Clara Medical Center, Santa Clara, California; 6 Department of Emergency Medicine, Kaiser Permanente Roseville Medical Center, Roseville, California

## Abstract

**Importance:**

Approximately 8% of acute pulmonary emboli are confined to the subsegmental arteries. The 2016 and 2021 American College of Chest Physicians (CHEST) guidelines and expert panel reports suggest the use of structured surveillance without anticoagulation for select ambulatory patients with subsegmental pulmonary embolism who do not have active cancer, deep vein thrombosis, impaired cardiopulmonary reserve, marked symptoms, or increased risk of recurrent venous thromboembolism; however, guideline uptake in community practice is unknown, as is the proportion of outpatients eligible for surveillance.

**Objective:**

To describe the prevalence of surveillance among outpatients with acute subsegmental pulmonary embolism and to estimate the proportion of patients eligible for structured surveillance using modified CHEST criteria.

**Design, Setting, and Participants:**

This retrospective cohort study was conducted across 21 US community hospitals in the Kaiser Permanente Northern California integrated health system from January 1, 2017, to December 31, 2021. Adult outpatients with acute subsegmental pulmonary embolism were included. Patients with the following higher-risk characteristics were excluded: codiagnoses requiring hospitalization, non–low-risk vital signs (ie, systolic blood pressure <90 mm Hg, pulse ≥110 bpm, or peripheral cutaneous pulse oximetry ≤92%), prediagnosis anticoagulant use, or hospice care. Data analysis was performed from November 2022 to February 2023.

**Main Outcomes and Measures:**

The main outcomes were the (1) prevalence of surveillance and (2) eligibility for surveillance using 2 sets of criteria: the CHEST criteria modified by excluding patients with higher-risk characteristics or right ventricular dysfunction and a stricter set of criteria requiring age younger than 65 years and no more than 1 embolus. The prevalence of structured surveillance was calculated and the proportion of patients eligible for surveillance was estimated.

**Results:**

Of the 666 outpatients with acute subsegmental pulmonary embolism included in this study, 229 with lower-risk characteristics were examined. Their median age was 58 (IQR, 42-68) years; more than half were men (120 [52.4%]) and self-identified as non-Hispanic White (128 [55.9%]). Six patients (2.6%) were initially not treated with anticoagulants. Among the lower-risk cohort, only 1 patient (0.4% [95% CI, 0.01%-2.4%]) underwent structured surveillance, without 90-day sequelae. Thirty-five patients (15.3% of the lower-risk group and 5.3% of the full cohort) were surveillance eligible using modified CHEST criteria. Fifteen patients (6.6% of the lower-risk group and 2.3% of the full cohort) were surveillance eligible using stricter criteria.

**Conclusions and Relevance:**

In this cohort study of lower-risk outpatients with subsegmental pulmonary embolism, few were eligible for structured surveillance, and only a small proportion of eligible patients underwent surveillance despite the CHEST guideline. If forthcoming trials find surveillance safe and effective, substantial uptake into clinical practice may require more than passive diffusion.

## Key Points

**Question:**

How prevalent is structured surveillance without anticoagulation for subsegmental pulmonary embolism in community practice, and what proportion of patients are surveillance eligible using modified American College of Chest Physicians (CHEST) criteria?

**Findings:**

In this cohort study of 666 outpatients examined over 5 years after publication of the 2016 CHEST guideline, only 1 patient (<1%) with subsegmental pulmonary embolism underwent surveillance without anticoagulation in a community setting with excellent follow-up access. Using modified CHEST criteria, 35 patients (5%) with subsegmental pulmonary embolism were surveillance eligible.

**Meaning:**

These findings suggest that only a small proportion of patients with subsegmental pulmonary embolism may be surveillance eligible, and structured surveillance is rarely used despite the CHEST guideline.

## Teaser

This cohort study examines the prevalence of and eligibility for structured surveillance of adult outpatients with acute subsegmental pulmonary embolism, according to modified American College of Chest Physicians criteria.

## Introduction

Pulmonary embolism (PE) confined to the subsegmental pulmonary arteries, known as subsegmental PE, has increased in prevalence as detection capacities of computed tomography pulmonary arteriography (CTPA) have improved. Approximately 3% to 12% of PEs diagnosed using CTPA are subsegmental.^1,2,3,4,5,6,7,8^ The pharmacological treatment of patients with low-risk subsegmental PE is contested.^4,9,10,11,12,13,14,15,16,17^ It is unclear whether the risk of progressive or recurrent venous thromboembolism (VTE) is sufficiently elevated to justify anticoagulation for all patients with subsegmental PE. The alternative to anticoagulation is a structured surveillance program, consisting of close follow-up, careful instructions for when to seek urgent medical attention, and serial lower-extremity compression ultrasonography to evaluate for deep vein thrombosis (DVT) on day 0 and again at 1 week.^14,18,19,20,21^ Would select patients with low-risk subsegmental PE be better served with structured surveillance without anticoagulation rather than assuming the risks, costs, and inconvenience associated with 3 to 6 months of anticoagulation? Previously, the safety and effectiveness of a surveillance strategy and its optimal candidates rested on limited and incomplete evidence. Recently, better evidence was published.^21^ No randomized clinical trials have yet been published to help direct clinicians in their pharmacotherapy decisions, but 2 are underway.^22,23^

The American College of Chest Physicians (CHEST) addressed this clinical conundrum in the 2016 CHEST guideline and expert panel report.^18^ The authors suggested structured surveillance without anticoagulation for ambulatory patients with stable subsegmental PE without active cancer, DVT (requiring bilateral compression ultrasonography regardless of DVT signs and symptoms), impaired cardiopulmonary reserve, marked symptoms, and increased risk of recurrent VTE. Kearon et al acknowledged that the “evidence supporting our recommendations is low quality because of indirectness and because there is limited ability to predict which patients will have VTE complications without anticoagulation.”^18^^(p339)^ This cautious recommendation was reiterated in the subsequent 2021 CHEST guideline and expert panel report.^19,20^ Although the eligibility criteria vary, the European Society of Cardiology and a multispecialty panel of VTE experts also recommend structured surveillance for select patients with low-risk subsegmental PE.^14,15,24^

The prevalence of surveillance strategies in community practice, even in settings with excellent follow-up, is unclear. The proportion of outpatients with acute subsegmental PE who would be eligible for structured surveillance is also unclear. We hypothesized that (1) structured surveillance, although suggested by CHEST, is uncommon in community clinical practice and (2) if the CHEST recommendations were followed, only a small proportion of patients with subsegmental PE would be eligible, given the numerous low-risk criteria required. Eligibility would shrink further if younger age (<65 years) and fewer clots (no more than 1) were required, based on the findings of the SSPE trial, a recent large, prospective observational study of structured surveillance.^21^

To test these 2 hypotheses, we undertook a cohort study of patients with lower-risk subsegmental PE to determine the prevalence of structured surveillance in a community-based setting and to ascertain hypothetical eligibility for surveillance based on modified CHEST criteria. Knowing how large of a difference the CHEST guideline has made (hypothesis 1) and could make (hypothesis 2) is the first step in understanding the application of structured surveillance without anticoagulation to outpatients with acute subsegmental PE.

## Methods

The Kaiser Permanente Northern California Institutional Review Board conducted a review of this retrospective cohort study and deemed it exempt from review and the requirement for informed consent under 45 CFR §46.104. The study was conducted in accordance with the principles of the Declaration of Helsinki.^25^ We followed the Strengthening the Reporting of Observational Studies in Epidemiology (STROBE) reporting guideline.

### Study Design and Setting

We performed this retrospective cohort study across 21 community medical centers and associated clinics of Kaiser Permanente Northern California. This health system cares for more than 4.5 million members, with more than 1.2 million emergency department (ED) visits per year. Kaiser Permanente health plan members have similar demographic and socioeconomic characteristics to the local and state populations.^26,27^

There were no systems or clinical decision supports in place for subsegmental PE, the management of which was at the discretion of the treating physicians, who had ready access to imaging studies, specialty consultation, and prompt follow-up.^3,28,29,30^ Patients who started oral anticoagulants (both warfarin and direct oral anticoagulants) were followed by a pharmacy-led, telephone-based anticoagulation management service.^31,32,33^

### Population

The study population consisted of all adult health plan members (aged ≥18 years) with CTPA demonstrating acute subsegmental PE from January 1, 2017, through December 31, 2021. We identified CTPA reports with a high probability of a positive subsegmental PE result using natural language processing algorithms (eMethods 1 in Supplement 1).

The computed tomography (CT) radiology reports identified as likely having a positive subsegmental PE result underwent manual medical record review. Patients were excluded for the following imaging reasons: negative, chronic, improving, or uncertain diagnosis of subsegmental PE, or PE not confined to the subsegmental arteries. We undertook manual review of the electronic health records of case patients with eligible CT radiology reports. To define the patient group with lower-risk characteristics, we excluded 437 case patients with a concomitant diagnosis requiring hospitalization (eg, severe COVID-19 pneumonia) (n = 253), with 1 or more non–low-risk vital signs (ie, systolic blood pressure <90 mm Hg, pulse ≥110 beats/min, or peripheral cutaneous pulse oximetry ≤92%) (n = 140), already taking anticoagulants^34^ (n = 41), or receiving hospice care (n = 3). The remaining patients constituted our lower-risk cohort (Figure).

**Figure.**
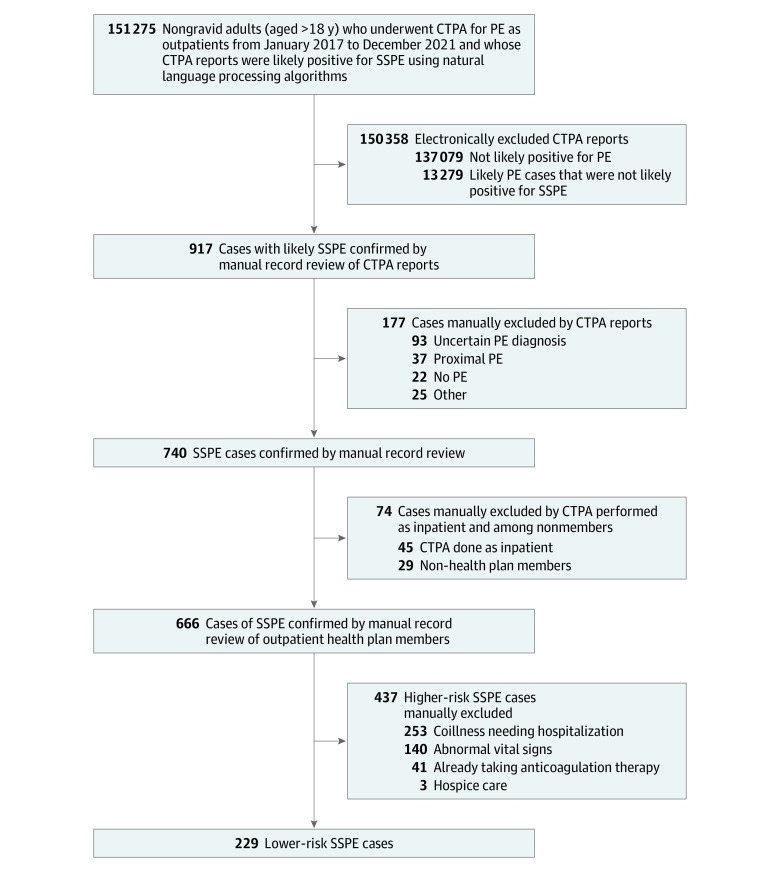
Cohort Assembly CT indicates computed tomography; CTPA, CT pulmonary angiography; PE, pulmonary embolism; SSPE, subsegmental PE.

### Outcomes

The first primary outcome was the prevalence of structured surveillance without anticoagulation. We defined surveillance as initially withholding anticoagulation, combined with clinical follow-up and DVT imaging scheduled within 2 weeks of the index PE diagnosis.

The second primary outcome was the prevalence of surveillance eligibility among 2 populations: those with lower-risk subsegmental PE (defined earlier in the Population section) and all patients with subsegmental PE. We determined surveillance eligibility using 2 sets of criteria: (1) those modified from the 2016 CHEST guideline and expert panel report^18^ and (2) enhanced criteria that also excluded older patients (aged ≥65 years) and those with more than 1 embolus, based on findings of the SSPE study.^21^

### Surveillance Criteria

The CHEST guideline and expert panel report published in 2016 (the year before the study period began) cautiously recommended structured surveillance without anticoagulation for select outpatients with stable acute subsegmental PE.^18^ We translated those recommendations into explicit surveillance eligibility criteria, as described in eTable 1 in Supplement 1. We assumed that patients without a documented recent provocation for VTE (eg, major surgery or oral estrogen) had none—a reasonable assumption in our setting, given our medical record review experience that clinicians nearly always inquire about and document recent VTE provocations for patients with acute PE.^2,3,30,35,36,37,38^ We deemed the absence of VTE provocation as a contraindication to withholding anticoagulation because of increased risk for VTE recurrence.^39^ We counted patients without compression ultrasonography as having a negative test result for DVT. We based this assumption on the multinational SSPE study, which identified concomitant DVT on serial ultrasonography in only 2% of patients with subsegmental PE and lower-risk attributes (eg, outpatients with stable disease and without cancer).^21^

We added several criteria to the CHEST recommendations, including pregnancy, clinical instability (based on non–low-risk vital signs), non-VTE diagnoses requiring inpatient care, and suggestions of right ventricular dysfunction (eTable 1 in Supplement 1).^40,41^ However, we did not require right ventricular assessment beyond CT evaluation: missing test results were categorized as though they were negative.^18^ Our criteria were admittedly incomplete, as some variables (eg, hemorrhage risk) were not available. As a sensitivity analysis, we required normal vital signs throughout the diagnostic evaluation (ie, lowest temperature ≥36 °C [or 96.8 °F], lowest systolic blood pressure ≥100 mm Hg, highest heart rate <100 beats/min, highest respiratory rate <20 breaths/min, and lowest pulse oximetry ≥95%). We compare our modified CHEST criteria with the criteria of 2 ongoing trials^22,23^ in eTable 2 in Supplement 1. We also captured standard 90-day outcomes, including major hemorrhage, recurrent VTE, and all-cause mortality (definitions in eMethods 2 in Supplement 1).

### Data Collection

Four physician abstractors (S.G.R., M.J.B., T.A., and D.R.V.) undertook manual medical record review after completing standardized training on data collection methods using a standardized computerized data collection tool, as in prior retrospective cohort studies of acute PE.^2^ The principal investigator (D.R.V.) answered abstraction questions throughout the study.

### Statistical Analysis

We examined characteristics including demographics (age, sex, and race and ethnicity), comorbidities, vital signs, laboratory results, and compression ultrasound results among patients with lower-risk subsegmental PE. Race and ethnicity were included to demonstrate that the diversity of the cohort reflects the diversity of the population of northern California and were self-reported as American Indian or Alaska Native, Asian or Pacific Islander, Black, Hispanic or Latino, or non-Hispanic White. We compared characteristics among 2 subcohorts: those who were ineligible and those who were eligible for structured surveillance without anticoagulation. We present categorical data as frequencies and proportions and continuous variables as medians (IQRs) or means (SDs). We report binomial exact 95% CIs where appropriate. All analyses were conducted with SAS, version 9.4 (SAS Institute Inc).

Of the lower-risk cases that underwent complete manual medical record review, we randomly selected 20% for independent review by a second physician abstractor (S.G.R. or D.R.V.) to assess interrater reliability. We report κ statistics and the percentage of agreement. Data analysis was performed from November 2022 to February 2023.

## Results

### Study Cohort

Of the 666 outpatients with acute subsegmental PE included in this study, we examined the 229 patients with lower-risk characteristics. A total of 120 patients were men (52.4%) and 109 were women (47.6%), with a median age of 58 (IQR, 42-68) years (Figure). With regard to race and ethnicity, 1 patient (0.4%) self-identified as American Indian or Alaska Native, 32 (14.0%) as Asian or Pacific Islander, 35 (15.3%) as Black, 33 (14.4%) as Hispanic or Latino, and 128 (55.9%) as non-Hispanic White. We report their characteristics and clinical evaluation in Table 1 and Table 2.

**Table 1.** Demographics and Characteristics of Patients With Lower-Risk Subsegmental Pulmonary Embolism, Stratified by Eligibility for Structured Surveillance Without Anticoagulation

Characteristic	No. of patients (%)		
	Lower-risk cohort (n = 229)	Surveillance eligibility^a^	
		No (n = 194)	Yes (n = 35)
Age, median (IQR), y	58 (42-68)	61 (46-70)	44 (34-60)
Sex			
Female	109 (47.6)	89 (45.9)	20 (57.1)
Male	120 (52.4)	105 (54.1)	15 (42.9)
Race and ethnicity			
American Indian or Alaska Native	1 (0.4)	1 (0.5)	0
Asian or Pacific Islander	32 (14.0)	28 (14.4)	4 (11.4)
Black	35 (15.3)	30 (15.5)	5 (14.3)
Hispanic or Latino	33 (14.4)	27 (13.9)	6 (17.1)
Non-Hispanic White	128 (55.9)	108 (55.7)	20 (57.1)
Comorbidity			
Obesity (BMI >30)	103 (45.0)	89 (45.9)	14 (40.0)
Hypertension	78 (34.1)	68 (35.1)	10 (28.6)
Chronic lung disease (includes asthma)	61 (26.6)	61 (31.4)	0
Obstructive sleep apnea	40 (17.5)	33 (17.0)	7 (20.0)
Prior VTE	37 (16.2)	37 (19.1)	0
Cancer, active	22 (9.6)	22 (11.3)	0
Heart failure (diastolic or systolic)	14 (6.1)	14 (7.2)	0
Phospholipid antibody	3 (1.3)	3 (1.6)	0
Crohn disease or ulcerative colitis	3 (1.3)	3 (1.6)	0
Factor V Leiden, homozygous	1 (0.4)	1 (0.5)	0
Charlson Comorbidity Index score^b^			
Mean (SD)	1.53 (2.1)	1.74 (2.1)	0.42 (1.3)
Median (IQR)	1 (0-2)	1 (0-3)	0
0	99 (43.2)	71 (36.6)	28 (80.0)
1	36 (15.7)	34 (17.5)	2 (5.7)
≥2	75 (32.8)	72 (37.1)	3 (8.6)
No measure (no visits in prior year)	19 (8.3)	17 (8.8)	2 (5.7)
VTE symptom^c^			
Thoracic pain	162 (70.7)	137 (70.6)	25 (71.4)
Dyspnea	154 (67.2)	130 (67.0)	24 (68.6)
Extremity pain or swelling	48 (21.0)	37 (19.1)	11 (31.4)
Palpitations	11 (4.8)	8 (4.1)	3 (8.6)
Syncope or presyncope	9 (3.9)	9 (4.6)	0
Hemoptysis	7 (3.1)	7 (3.6)	0
None	2 (0.9)	2 (1.0)	0
VTE symptom duration			
<48 h	96 (41.9)	77 (39.7)	19 (54.3)
≥48 h to <7 d	72 (31.4)	62 (32.0)	10 (28.6)
≥7 to <14 d	28 (12.2)	22 (11.3)	6 (17.1)
≥14 to <30 d	16 (7.0)	16 (8.2)	0
≥30 to <60 d	6 (2.6)	6 (3.1)	0
≥60 d	8 (3.5)	8 (4.1)	0
Unclear	1 (0.4)	1 (0.5)	0
PE Severity Index class^d^			
I	82 (35.8)	62 (32.0)	20 (57.1)
II	58 (25.3)	51 (26.3)	7 (20.0)
III	49 (21.4)	42 (21.7)	7 (20.0)
IV	25 (10.9)	25 (12.9)	0
V	9 (3.9)	9 (4.6)	0
Unable to calculate	6 (2.6)	5 (2.6)	1 (2.9)

Abbreviations: BMI, body mass index (calculated as weight in kilograms divided by height in meters squared); PE, pulmonary embolism; VTE, venous thromboembolism.

^a^ Criteria definitions are presented in eTable 1 in Supplement 1.

^b^ The Charlson Comorbidity Index is a method of categorizing comorbidities of patients. Each comorbidity category has an associated weight, based on the adjusted risk of mortality or resource use, and the sum of all the weights results in a single comorbidity score for a patient. A score of 0 indicates that no comorbidities were found.

^c^ Patients often reported more than 1 VTE symptom.

^d^ The PE Severity Index is a widely used, validated index to predict risk of 30-day all-cause mortality in patients with acute PE. The index is composed of 11 weighted clinical variables and stratifies patients into 5 risk classes, each higher class associated with an ascending incidence of 30-day all-cause mortality. This score was unable to be calculated for 6 patients due to a lack of results for complete vital sign variables.

**Table 2.** Vital Sign, Laboratory, and Compression Ultrasound Results for Patients With Lower-Risk Subsegmental Pulmonary Embolism, Stratified by Eligibility for Structured Surveillance Without Anticoagulation

Characteristic	No. of patients (%)		
	Lower-risk cohort (n = 229)	Surveillance eligibility^a^	
		No (n = 194)	Yes (n = 35)
Vital sign^b^			
Systolic blood pressure, mm Hg			
≥100	210 (91.7)	180 (92.8)	30 (85.7)
<100 and ≥90	13 (5.7)	9 (4.6)	4 (11.4)
Missing	6 (2.6)	5 (2.6)	1 (2.9)
Pulse, beats/min			
<80	75 (32.8)	61 (31.4)	14 (40.0)
≥80 and <100	116 (50.7)	101 (52.1)	15 (42.9)
≥100 and <110	30 (13.1)	25 (12.9)	5 (14.3)
Missing	8 (3.5)	7 (3.6)	1 (2.9)
Respiratory rate, breaths/min			
<24	166 (72.5)	141 (72.7)	25 (71.4)
≥24 and <30	24 (10.5)	21 (10.8)	3 (8.6)
≥30	3 (1.3)	3 (1.6)	0
Missing	36 (15.7)	29 (15.0)	7 (20.0)
Oxygen saturation, %			
≥95	193 (84.3)	161 (83.0)	32 (91.4)
93-94	29 (12.7)	27 (13.9)	2 (5.7)
Missing	7 (3.1)	6 (3.1)	1 (2.9)
Laboratory value			
Troponin, ng/mL			
Normal (0-0.04)	183 (79.9)	153 (78.9)	30 (85.7)
Abnormal (>0.04)	8 (3.5)	8 (4.1)	0
Not measured	38 (16.6)	33 (17.0)	5 (14.3)
B-type natriuretic peptide, pg/mL			
<100	78 (34.1)	66 (34.0)	12 (34.3)
≥100 < 500	21 (9.2)	20 (10.3)	1 (2.9)
≥500	4 (1.8)	4 (2.1)	0
Not measured	126 (55.0)	104 (53.6)	22 (62.9)
Deep vein thrombosis			
Negative	54 (23.6)	41 (21.1)	13 (37.2)
Positive	20 (8.7)	20 (10.3)	0
Not measured	155 (67.7)	133 (68.6)	22 (62.9)

SI conversion factor: To convert troponin to μg/L, multiply by 1.0.

^a^ Criteria definitions are presented in eTable 1 in Supplement 1.

^b^ The most abnormal vital sign recorded during the index encounter(s) in the direction in question: lowest systolic blood pressure, highest pulse, highest respiratory rate, or lowest oxygen saturation.

### General Patient Care

Overall, the majority of patients (172 [75.1%]) with lower-risk subsegmental PE were initially evaluated in the ED, whereas the remaining 57 (24.9%) were initially evaluated in the clinic. Sixteen clinic patients (28.1%) were treated without referral to the ED or hospital.^30^ Among the 213 patients treated in the ED, most (142 [66.7%]) were discharged home directly from the ED; 25 ED patients (11.7%) were admitted to a short-stay outpatient observation area (20 of whom were discharged home from the unit) and 51 patients (23.9%) were admitted from the ED to the hospital. Treating physicians commonly consulted specialists (140 [61.1%]) to discuss PE management, most frequently adult hospitalists (96 of 140 [41.9%]).

Among the 229 patients in the lower-risk cohort, 223 (97.4%) received anticoagulation initially. The most common anticoagulant prescribed was rivaroxaban (112 [50.2%]) (eTable 3 in Supplement 1). After the initial diagnostic encounter, 173 patients (75.5%) had follow-up with their clinicians within 7 days. In addition, 203 patients (88.6%) had follow-up with the anticoagulation management service within 7 days.

### Study Outcomes

We identified only 1 patient (0.4% [95% CI, 0.01%-2.4%]) among our lower-risk cohort who underwent a guideline-recommended regimen of structured surveillance, receiving repeat compression ultrasonography of the lower extremities 9 days after the index diagnosis. This patient did not experience recurrent VTE or major hemorrhage within 90 days. The surveilled patient was 1 of 6 patients (2.6%) who were not initially anticoagulated upon discharge home (eTable 4 in Supplement 1). Two of these patients met our modified CHEST criteria for surveillance, and none met our enhanced criteria. Three patients who had not received anticoagulation were started on anticoagulants upon follow-up with their respective primary care physicians, each of whom had consulted specialists for treatment advice.^15^ Two patients remained without anticoagulation without receiving surveillance imaging within 2 weeks; one of these patients was scheduled for a follow-up CTPA, but the imaging study was not completed.

Of the 229 patients with lower-risk characteristics, 35 (15.3%) were eligible for structured surveillance using modified CHEST criteria (eTable 1 in Supplement 1), representing 5.3% of the full subsegmental PE cohort of 666 patients. After we applied the enhanced criteria (by adding age and clot number limitations), only 15 patients (6.6%) with lower-risk characteristics were eligible for surveillance, representing 2.3% of the full cohort (Table 3). We also report eligibility results in Table 3 if normal vital signs were required for structured surveillance.

**Table 3.** Percentage of Patients With Acute Subsegmental Pulmonary Embolism Eligible for Structured Surveillance Without Anticoagulation Based on Different Eligibility Criteria, Stratified by Risk Group

Surveillance eligibility criterion	No. of patients	Percentage eligible by risk group	
		Lower-risk cohort (n = 229)^a^	Full cohort (N = 666)
1. Modified 2016 CHEST criteria	35	15.3	5.3
Restricted to age <65 y and no more than 1 embolus	15	6.6	2.3
2. Modified 2016 CHEST criteria reduced by requiring strictly normal vital signs	13	5.7	2.0
Restricted to age <65 y and no more than 1 embolus	6	2.6	0.9

Abbreviation: CHEST, American College of Chest Physicians.

^a^ Criteria definitions are presented in eTable 1 in Supplement 1.

Concomitant DVT, active cancer, and pregnancy are agreed on as contraindications to surveillance for patients with subsegmental PE (eTable 2 in Supplement 1). Other modified CHEST criteria are missing from the ongoing trials^22,23^; for example, the trials did not exclude from surveillance patients whose index PE was not provoked by a reversible risk factor (eg, recent surgery) (eTable 2 in Supplement 1). Patients with unprovoked PE, however, are at increased risk for VTE recurrence compared with those who have a transient, reversible risk factor,^39^ which is why some recommend excluding them from surveillance. We quantified the difference in surveillance eligibility if these debated criteria were removed as surveillance exclusions (eTable 5 in Supplement 1). For example, if patients with impaired cardiopulmonary reserve were not excluded from surveillance, then the number of eligible case patients would increase by 17, from 35 (5.3%) to 52 (7.8%). Removing all debated categories would increase the number eligible by 83, from 35 (5.3%) to 118 (17.7%) of the 666 patients with subsegmental PE.

In Tables 1 and 2, we report characteristics of patients who were and were not eligible for structured surveillance without anticoagulation using our modified CHEST criteria. Compared with patients who were ineligible, the surveillance-eligible group was younger, had a lower frequency of comorbidities, and had a greater proportion of patients with low-risk PE (classes I and II of the PE Severity Index). The surveillance-eligible group also tended to have a higher proportion of patients with normal vital signs and test results compared with their surveillance-ineligible counterparts. Few patients had no VTE-related symptoms.^42^

Overall, at 90 days, 1 of the 229 patients (0.4%) with lower-risk characteristics had received anticoagulants and had a nonfatal case of major hemorrhage. No patients with lower-risk characteristics had recurrent VTE. Three patients died (1.3%) from preexisting comorbidities other than VTE.

The κ values and percentage of agreement, respectively, were as follows: meets lower-risk criteria (κ not calculated; 100%), number of PEs (κ = 1.0; 100%), chronic lung disease (κ = 1.0; 100.0%), maximum respiratory rate (κ = 0.90; 95.7%), and anticoagulation at index encounter (κ = 0.94; 97.8%). κ values between 0.81 and 1.00 indicate almost perfect agreement. κ values were not calculated for the variable meets lower-risk criteria because the 2 raters assigned every patient the “yes” value.

## Discussion

In this retrospective cohort study of community hospitals in the Kaiser Permanente Northern California integrated health system, we observed that the prevalence of structured surveillance without anticoagulation for outpatients with lower-risk acute subsegmental PE over 5 years was remarkably low (<1%).^15^ These findings suggest that there was almost no effect on surveillance practices attributable to the CHEST guideline in this large US health care system—a system that is conducive to structured surveillance, with ready access to VTE imaging, specialty consultation, and timely primary care follow-up.^2,3,28,38,43^

Reasons for the limited uptake are likely multifactorial. To start, the exclusion criteria are extensive, making the pool of eligible patients small, as we observed. This alone would make implementation of the guideline recommendations challenging without a system-based strategy of implementation, such as a clinical decision support tool integrated with the electronic health record.^3^ In addition, specialty recommendations may be unfamiliar to primary care and emergency medicine clinicians who diagnose most cases of outpatient subsegmental PE in the US. Our 1 case of structured surveillance illustrates this point, as the medical decision-making was undertaken by a pulmonologist, not a generalist. Moreover, surveillance contravenes the long-established convention of anticoagulating PE unless contraindicated. Well-established practice patterns can be difficult to overturn, as attested by the slow and sporadic uptake of outpatient PE management in the US.^44,45^ If forthcoming trials of structured surveillance without anticoagulation support the practice among a select cohort of outpatients, a concerted effort will be needed to translate the science of surveillance into routine clinical practice.^3,38^

We estimated that few outpatients (35 [5.3%]) with acute subsegmental PE in this study would have been eligible for structural surveillance without anticoagulation using our modified CHEST criteria. The proportion eligible for surveillance diminished further when age and clot number criteria were also required. We estimated that removing these enhanced restrictions as well as the debated elements of our modified CHEST criteria would have increased the percentage to 17.7%.

In the larger PE population, subsegmental cases are relatively uncommon (approximately 8%). From our results, we estimated that only 2% to 18% of subsegmental cases would meet criteria for structured surveillance. If these 2 prevalence estimates were combined, only a small fraction (<2%) of all outpatients with acute PE would be surveillance eligible. Furthermore, the complexity of the CHEST surveillance eligibility criteria paired with ensuring timely follow-up imaging would make it difficult for many primary care and emergency medicine clinicians to identify and apply structured surveillance in practice. Based on the current literature, we think it prudent to treat patients with subsegmental PE like those with a more proximal PE: anticoagulate unless contraindicated. However, we recognize that certain patient subpopulations (eg, athletes who participate in contact or extreme sports) may be at higher risk of bleeding complications from anticoagulation and might benefit from structured surveillance without anticoagulation. Particularly in these select situations, a patient-tailored approach grounded in shared decision-making of risks, benefits, and patient preferences is warranted.

Comparison of the proportion of patients with acute subsegmental PE who we identified as eligible for structured surveillance with other studies is difficult. The few studies that used a protocol to identify surveillance-eligible patients did not report sufficient detail about their excluded population to allow comparison.^21,46^ For example, our most common exclusion (in one-third of patients) was for concomitant diagnoses that required inpatient care (eg, severe COVID-19 pneumonia or decompensated heart failure). Such patients were not assessed for study eligibility in the SSPE study, as they could not have been transferred safely from the ED to the thrombosis clinic for treatment, which was required by the protocol.^21^

### Limitations

This study has limitations inherent to its retrospective nature, which we attempted to mitigate by adhering to established guidelines for medical record review–based studies.^47,48^ Physician abstractors, however, were not blinded to patient variables when identifying study outcomes, which is a possible source of bias. Our method of case ascertainment was incomplete, as it depended on radiology CTPA reports, which do not always document the location of PEs that are confined to the subsegmental arteries.^2^ Our approach to surveillance was conservative. The proportion of eligible patients will vary as criteria change, as we observed. Our sample size was limited to a 5-year period, given resource constraints. Because the study population comprised only northern California residents, the results of this study may not be generalizable to other locations and practice settings.

## Conclusions

In this cohort study of lower-risk outpatients with subsegmental PE, we observed that the 2016 CHEST recommendation for structured surveillance without anticoagulation was rarely used over the subsequent 5 years. This study was conducted in a health care setting conducive to structured surveillance, with ready access to VTE imaging, specialty consultation, and timely follow-up with primary care. We also observed that our modified CHEST criteria for structured surveillance would have identified only a small proportion of outpatients with subsegmental PE who were eligible for surveillance. Although trials are ongoing to define which patients with subsegmental PE can safely undergo surveillance, widespread uptake of any new surveillance practice will require more than passive diffusion.

## Notes

Supplement 1. **eMethods 1.** Development of a Natural Language Processing Algorithm to Identity Radiology Reports of Comsputed Tomography Pulmonary Angiography Likely Positive for Subsegmental Pulmonary Embolism **eTable 1.** Conditions That Favor Anticoagulation Over Structured Surveillance for Patients With Subsegmental Pulmonary Embolism Translated Into Explicit Surveillance Exclusion Criteria **eTable 2.** Excluding Patients With Subsegmental Pulmonary Embolism From Structured Surveillance Without Anticoagulation: Comparing Our Modified CHEST Criteria With Criteria of 2 Ongoing Trials **eMethods 2.** Definitions of 90-Day Outcomes **eTable 3.** Initial Anticoagulation Treatment for Patients With Lower-Risk Acute Subsegmental Pulmonary Embolism **eTable 4.** Case Patients With Subsegmental Pulmonary Embolism Initially Treated Without Anticoagulation **eTable 5.** Exclusion Criteria for Structured Surveillance Without Anticoagulation for Patients With Lower-Risk Acute Subsegmental Pulmonary Embolism and Revision of Surveillance Eligibility if Criterion Removed
eReferences

Supplement 2.
Data Sharing Statement

## References

[1] Carrier M, Righini M, Wells PS, . Subsegmental pulmonary embolism diagnosed by computed tomography: incidence and clinical implications. A systematic review and meta-analysis of the management outcome studies. J Thromb Haemost. 2010;8(8):1716-1722. doi:10.1111/j.1538-7836.2010.03938.x 20546118

[2] Vinson DR, Ballard DW, Huang J, ; MAPLE Investigators of the KP CREST Network. Outpatient management of emergency department patients with acute pulmonary embolism: variation, patient characteristics, and outcomes. Ann Emerg Med. 2018;72(1):62-72.e3. doi:10.1016/j.annemergmed.2017.10.022 29248335

[3] Vinson DR, Mark DG, Chettipally UK, ; eSPEED Investigators of the KP CREST Network. Increasing safe outpatient management of emergency department patients with pulmonary embolism: a controlled pragmatic trial. Ann Intern Med. 2018;169(12):855-865. doi:10.7326/M18-1206 30422263

[4] Fernández-Capitán C, Rodriguez Cobo A, Jiménez D, ; RIETE Investigators. Symptomatic subsegmental versus more central pulmonary embolism: clinical outcomes during anticoagulation. Res Pract Thromb Haemost. 2020;5(1):168-178. doi:10.1002/rth2.12446 33537541 PMC7845079

[5] Dahan A, Farina S, Holmes NE, . Subsegmental pulmonary embolism and anticoagulant therapy: the impact of clinical context. Intern Med J. Published online May 2, 2022. doi:10.1111/imj.15789 35499105

[6] Armitage MN, Mughal AZ, Huntley CC, Lasserson D, Newnham M. A multicentre observational study of the prevalence, management, and outcomes of subsegmental pulmonary embolism. J Thromb Thrombolysis. 2023;55(1):126-133. doi:10.1007/s11239-022-02714-5 36342637 PMC9925472

[7] Becattini C, Agnelli G, Maggioni AP, ; COPE Investigators. Contemporary management and clinical course of acute pulmonary embolism: the COPE study. Thromb Haemost. 2023;123(6):613-626. doi:10.1055/a-2031-3859 36758612 PMC10205399

[8] Roussel M, Bloom B, Taalba M, ; Improving Emergency Care (IMPEC) FHU Collaborator Group. Temporal trends in the use of computed tomographic pulmonary angiography for suspected pulmonary embolism in the emergency department: a retrospective analysis. Ann Intern Med. 2023;176(6):761-768. doi:10.7326/M22-3116 37216659

[9] Bariteau A, Stewart LK, Emmett TW, Kline JA. Systematic review and meta-analysis of outcomes of patients with subsegmental pulmonary embolism with and without anticoagulation treatment. Acad Emerg Med. 2018;25(7):828-835. doi:10.1111/acem.13399 29498138

[10] Raslan IA, Chong J, Gallix B, Lee TC, McDonald EG. Rates of overtreatment and treatment-related adverse effects among patients with subsegmental pulmonary embolism. JAMA Intern Med. 2018;178(9):1272-1274. doi:10.1001/jamainternmed.2018.2971 30073241 PMC6142970

[11] Moores LK. Are we overtreating isolated subsegmental pulmonary embolism? First do no harm. JAMA Intern Med. 2018;178(9):1274-1275. doi:10.1001/jamainternmed.2018.2970 30073302

[12] Roberge G, Carrier M. How to manage patients with symptomatic subsegmental pulmonary embolism? Pol Arch Intern Med. 2020;130(4):310-316. doi:10.20452/pamw.15211 32091505

[13] Yoo HH, Nunes-Nogueira VS, Fortes Villas Boas PJ. Anticoagulant treatment for subsegmental pulmonary embolism. Cochrane Database Syst Rev. 2020;2(2):CD010222. 32030721 10.1002/14651858.CD010222.pub4 PMC7004894

[14] den Exter PL, Kroft LJM, Gonsalves C, . Establishing diagnostic criteria and treatment of subsegmental pulmonary embolism: a Delphi analysis of experts. Res Pract Thromb Haemost. 2020;4(8):1251-1261. doi:10.1002/rth2.12422 33313465 PMC7695556

[15] Vinson DR, Isaacs DJ, Taye E, Balasubramanian MJ. Challenges in managing isolated subsegmental pulmonary embolism. Perm J. 2021;25:21.077. doi:10.7812/TPP/21.077 35348105 PMC8784082

[16] Westafer LM, Vinson DR. Risk for recurrent venous thromboembolism in patients with subsegmental pulmonary embolism managed without anticoagulation. Ann Intern Med. 2022;175(4):W43. doi:10.7326/L22-0037 35436438

[17] Parsirad M, Rahimi B, Peiman S, Zebardast J, Zangene E. A survey of physicians’ opinions about the treatment of subsegmental pulmonary embolism. Can J Respir Ther. 2022;58:53-56. doi:10.29390/cjrt-2021-053 35509977 PMC9020570

[18] Kearon C, Akl EA, Ornelas J, . Antithrombotic therapy for VTE disease: CHEST guideline and expert panel report. Chest. 2016;149(2):315-352. doi:10.1016/j.chest.2015.11.026 26867832

[19] Stevens SM, Woller SC, Baumann Kreuziger L, . Executive summary: antithrombotic therapy for VTE disease: second update of the CHEST guideline and expert panel report. Chest. 2021;160(6):2247-2259. doi:10.1016/j.chest.2021.07.056 34352279

[20] Stevens SM, Woller SC, Kreuziger LB, . Antithrombotic therapy for VTE disease: second update of the CHEST guideline and expert panel report. Chest. 2021;160(6):e545-e608. doi:10.1016/j.chest.2021.07.055 34352278

[21] Le Gal G, Kovacs MJ, Bertoletti L, ; SSPE Investigators. Risk for recurrent venous thromboembolism in patients with subsegmental pulmonary embolism managed without anticoagulation: a multicenter prospective cohort study. Ann Intern Med. 2022;175(1):29-35. doi:10.7326/M21-2981 34807722

[22] Clinical surveillance vs. anticoagulation for low-risk patients with isolated subsegmental pulmonary embolism (SAFE-SSPE). ClinicalTrials.gov identifier: NCT04263038. Updated February 23, 2023. Accessed June 30, 2023. https://clinicaltrials.gov/study/NCT04263038

[23] Stopping anticoagulation for isolated or incidental subsegmental pulmonary embolism (STOPAPE). ClinicalTrials.gov identifier: NCT04727437. Updated September 29, 2021. Accessed June 30, 2023. https://clinicaltrials.gov/study/NCT04727437

[24] Konstantinides SV, Meyer G, Becattini C, ; ESC Scientific Document Group. 2019 ESC guidelines for the diagnosis and management of acute pulmonary embolism developed in collaboration with the European Respiratory Society (ERS). Eur Heart J. 2020;41(4):543-603. doi:10.1093/eurheartj/ehz405 31504429

[25] World Medical Association. World Medical Association Declaration of Helsinki: ethical principles for medical research involving human subjects. JAMA. 2013;310(20):2191-2194. doi:10.1001/jama.2013.281053 24141714

[26] Gordon N, Lin T. The Kaiser Permanente Northern California Adult Member Health Survey. Perm J. 2016;20(4):15-225. doi:10.7812/TPP/15-225 27548806 PMC5101088

[27] Davis AC, Voelkel JL, Remmers CL, Adams JL, McGlynn EA. Comparing Kaiser Permanente members to the general population: implications for generalizability of research. Perm J. 2023;27(2):87-98. doi:10.7812/TPP/22.172 37170584 PMC10266863

[28] Vinson DR, Ballard DW, Huang J, Rauchwerger AS, Reed ME, Mark DG; Kaiser Permanente CREST Network. Timing of discharge follow-up for acute pulmonary embolism: retrospective cohort study. West J Emerg Med. 2015;16(1):55-61. doi:10.5811/westjem.2014.12.23310 25671009 PMC4307727

[29] Vinson DR, Mark DG, Ballard DW. Outpatient management of patients with pulmonary embolism. Ann Intern Med. 2019;171(3):228. doi:10.7326/L19-0208 31382285

[30] Vinson DR, Hofmann ER, Johnson EJ, ; PEPC Investigators of the KP CREST Network. Management and outcomes of adults diagnosed with acute pulmonary embolism in primary care: community-based retrospective cohort study. J Gen Intern Med. 2022;37(14):3620-3629. doi:10.1007/s11606-021-07289-0 35020167 PMC9585133

[31] An J, Niu F, Zheng C, . Warfarin management and outcomes in patients with nonvalvular atrial fibrillation within an integrated health care system. J Manag Care Spec Pharm. 2017;23(6):700-712. doi:10.18553/jmcp.2017.23.6.700 28530526 PMC10398296

[32] Sylvester KW, Ting C, Lewin A, . Expanding anticoagulation management services to include direct oral anticoagulants. J Thromb Thrombolysis. 2018;45(2):274-280. doi:10.1007/s11239-017-1602-1 29274044

[33] Clark NP. Role of the anticoagulant monitoring service in 2018: beyond warfarin. Hematology Am Soc Hematol Educ Program. 2018;2018(1):348-352. doi:10.1182/asheducation-2018.1.348 30504331 PMC6246023

[34] Liu MY, Ballard DW, Huang J, . Acute pulmonary embolism in emergency department patients despite therapeutic anticoagulation. West J Emerg Med. 2018;19(3):510-516. doi:10.5811/westjem.2018.1.35586 29760849 PMC5942018

[35] Vinson DR, Engelhart DC, Bahl D, . Presyncope is associated with intensive care unit admission in emergency department patients with acute pulmonary embolism. West J Emerg Med. 2020;21(3):703-713. doi:10.5811/westjem.2020.2.45028 32421523 PMC7234693

[36] Vinson DR, Drenten CE, Huang J, ; Kaiser Permanente Clinical Research on Emergency Services and Treatment (CREST) Network. Impact of relative contraindications to home management in emergency department patients with low-risk pulmonary embolism. Ann Am Thorac Soc. 2015;12(5):666-673. doi:10.1513/AnnalsATS.201411-548OC 25695933 PMC4743639

[37] Ballard DW, Vemula R, Chettipally UK, ; KP CREST Network Investigators. Optimizing clinical decision support in the electronic health record. Clinical characteristics associated with the use of a decision tool for disposition of ED patients with pulmonary embolism. Appl Clin Inform. 2016;7(3):883-898. doi:10.4338/ACI-2016-05-RA-0073 27652375 PMC5052556

[38] Vinson DR, Casey SD, Vuong PL, Huang J, Ballard DW, Reed ME. Sustainability of a clinical decision support intervention for outpatient care for emergency department patients with acute pulmonary embolism. JAMA Netw Open. 2022;5(5):e2212340. doi:10.1001/jamanetworkopen.2022.12340 35576004 PMC9112064

[39] Kearon C, Ageno W, Cannegieter SC, Cosmi B, Geersing GJ, Kyrle PA; Subcommittees on Control of Anticoagulation, and Predictive and Diagnostic Variables in Thrombotic Disease. Categorization of patients as having provoked or unprovoked venous thromboembolism: guidance from the SSC of ISTH. J Thromb Haemost. 2016;14(7):1480-1483. doi:10.1111/jth.13336 27428935

[40] Becattini C, Maraziti G, Vinson DR, . Right ventricle assessment in patients with pulmonary embolism at low risk for death based on clinical models: an individual patient data meta-analysis. Eur Heart J. 2021;42(33):3190-3199. doi:10.1093/eurheartj/ehab329 34179965

[41] Maraziti G, Vinson DR, Becattini C. Echocardiography for risk stratification in patients with pulmonary embolism at low risk of death: a response. Eur Heart J. 2021;43(1):86-87. doi:10.1093/eurheartj/ehab779 34788408

[42] Rodríguez-Cobo A, Fernández-Capitán C, Tung-Chen Y, ; The Riete Investigators. Clinical significance and outcome in patients with asymptomatic versus symptomatic subsegmental pulmonary embolism. J Clin Med. 2023;12(4):1640. doi:10.3390/jcm12041640 36836176 PMC9959177

[43] Casey SD, Zekar L, Somers MJ, Westafer LM, Reed ME, Vinson DR. Embolism laterality and highest heart rate predict hospitalization of emergency department patients with acute, low-risk pulmonary embolism. Ann Emerg Med. 2023;S0196-0644(23)00123-3. doi:10.1016/j.annemergmed.2023.02.014 37028997 PMC11126867

[44] Westafer LM, Shieh MS, Pekow PS, Stefan MS, Lindenauer PK. Outpatient management of patients following diagnosis of acute pulmonary embolism. Acad Emerg Med. 2021;28(3):336-345. doi:10.1111/acem.14181 33248008 PMC8221072

[45] Westafer LM, Jessen E, Boccio E, . Barriers and facilitators to the outpatient management of low-risk pulmonary embolism from the emergency department. Ann Emerg Med. Published online April 12, 2023. doi:10.1016/j.annemergmed.2023.02.021 PMC10440853 37596016

[46] Mehta D, Barnett M, Zhou L, . Management and outcomes of single subsegmental pulmonary embolus: a retrospective audit at North Shore Hospital, New Zealand. Intern Med J. 2014;44(9):872-876. doi:10.1111/imj.12507 24942202

[47] Gilbert EH, Lowenstein SR, Koziol-McLain J, Barta DC, Steiner J. Chart reviews in emergency medicine research: where are the methods? Ann Emerg Med. 1996;27(3):305-308. doi:10.1016/S0196-0644(96)70264-0 8599488

[48] Kaji AH, Schriger D, Green S. Looking through the retrospectoscope: reducing bias in emergency medicine chart review studies. Ann Emerg Med. 2014;64(3):292-298. doi:10.1016/j.annemergmed.2014.03.025 24746846

