# Supplementary material for: Prevalence of and Eligibility for Surveillance Without Anticoagulation Among Adults With Lower-Risk Acute Subsegmental Pulmonary Embolism
Source: JAMA Netw Open. 2023 Aug 2;6(8):e2326898. doi: 10.1001/jamanetworkopen.2023.26898 (PMC10398409; doi:10.1001/jamanetworkopen.2023.26898)
Supplement: Supplement 1. — eMethods 1. Development of a Natural Language Processing Algorithm to Identity Radiology Reports of Comsputed Tomography Pulmonary Angiography Likely Positive for Subsegmental Pulmonary Embolism eTable 1. Conditions That Favor Anticoagulation Over Structured Surveillance for Patients With Subsegmental Pulmonary Embolism Translated Into Explicit Surveillance Exclusion Criteria eTable 2. Excluding Patients With Subsegmental Pulmonary Embolism From Structured Surveillance Without Anticoagulation: Comparing Our Modified CHEST Criteria With Criteria of 2 Ongoing Trials eMethods 2. Definitions of 90-Day Outcomes eTable 3. Initial Anticoagulation Treatment for Patients With Lower-Risk Acute Subsegmental Pulmonary Embolism eTable 4. Case Patients With Subsegmental Pulmonary Embolism Initially Treated Without Anticoagulation eTable 5. Exclusion Criteria for Structured Surveillance Without Anticoagulation for Patients With Lower-Risk Acute Subsegmental Pulmonary Embolism and Revision of Surveillance Eligibility if Criterion Removed eReferences [file jamanetwopen-e2326898-s001.pdf]

Supplemental Online Content

Rouleau SG, Balasubramanian MJ, Huang J, Antognini T, Reed ME, Vinson DR. Prevalence of and eligibility for surveillance without anticoagulation among adults with lower-risk acute subsegmental pulmonary embolism. *JAMA Netw Open*. 2023;6(8):e2326898. doi:10.1001/jamanetworkopen.2023.26898

eMethods 1. Development of a Natural Language Processing Algorithm to Identity Radiology Reports of Computed Tomography Pulmonary Angiography Likely Positive for Subsegmental Pulmonary Embolism

eTable 1. Conditions That Favor Anticoagulation Over Structured Surveillance for Patients With Subsegmental Pulmonary Embolism Translated Into Explicit Surveillance Exclusion Criteria

eTable 2. Excluding Patients With Subsegmental Pulmonary Embolism From Structured Surveillance Without Anticoagulation: Comparing Our Modified CHEST Criteria With Criteria of 2 Ongoing Trials

eMethods 2. Definitions of 90-Day Outcomes

eTable 3. Initial Anticoagulation Treatment for Patients With Lower-Risk Acute Subsegmental Pulmonary Embolism

eTable 4. Case Patients With Subsegmental Pulmonary Embolism Initially Treated Without Anticoagulation

eTable 5. Exclusion Criteria for Structured Surveillance Without Anticoagulation for Patients With Lower-Risk Acute Subsegmental Pulmonary Embolism and Revision of Surveillance Eligibility if Criterion Removed

eReferences

This supplemental material has been provided by the authors to give readers additional information about their work.

© 2023 Rouleau SG et al. *JAMA Network Open*.

eMethods 1. Development of a Natural Language Processing Algorithm to Identity Radiology Reports of Computed Tomography Pulmonary Angiography Likely Positive for Subsegmental Pulmonary Embolism

We used natural language processing (NLP) algorithms developed for a separate study[50] to identify computed tomography (CT) pulmonary angiography radiology reports that were likely positive for pulmonary embolism. In that study, colleagues from our CREST Network manually reviewed a training dataset of 700 CT reports to identify tomograms performed to evaluate for pulmonary embolism and, if ordered to diagnose pulmonary embolism, then to identify positive or negative pulmonary embolism findings. This dataset was used to develop and iteratively refine 2 NLP algorithms: the first to identify CTs undertaken to diagnose pulmonary embolism (NLP1) and the second to identify CTs positive for pulmonary embolism (NLP2). Once both NLP algorithms had been finalized, they applied them to the remaining CT reports and reviewed an additional 1,500 reports (500 for NLP1 and 1000 for NLP2) to validate NLP performance. To establish inter-rater reliability, a random sample of 10% of the NLP derivation dataset was reviewed by 3 investigators. Against manual report review as the criterion standard, the NLP algorithms used in tandem demonstrated 93% sensitivity and 98% specificity in identifying pulmonary embolism, with positive predictive value of 85% and negative predictive value of 99%. The area under the receiver operator characteristic curve was 0.96 for correctly identifying negative CT pulmonary angiography studies (Wald 95% confidence interval [CI] 0.93 to 0.98). Interrater reliability kappa scores were 0.93 (95% CI 0.79-1.0) for NLP1 and 1.0 (perfect agreement) for NLP2. We went on to use these NLP algorithms in other pulmonary embolism studies.[35,41]

For this current study, we used the above NLP algorithms to screen 151,275 CT reports and identified 14,196 (9.4%) that were likely positive for pulmonary embolism. We then developed a text string algorithm using an iterative process to identify the subcohort of reports with probable subsegmental pulmonary embolism. We searched for cases with ‘filling defects?|emboli|embolism|embolus|thrombi|thrombus|clot) (up to 8 words) subsegmental’ or ‘subsegmental (up to 6 words) (embolus|embolism|emboli|thromboemboli|nonocculusive clot|filling defects?|foci of PE|PE)’ to identify. We excluded cases with ‘segmental (up to 2 words) subsegmental’ or ‘extending (up to 3 words) subsegmental’ or ‘saddle embolus/embolism/emboli’ or ‘not excluded at the subsegmental artery level’. We identified 917 (6.5%) of 14,196 reports that were likely positive for subsegmental pulmonary embolism. Study physicians then manually reviewed these 917 CT reports for criteria of study eligibility (Figure 1).

© 2023 Rouleau SG et al. *JAMA Network Open*.

eTable 1. Conditions That Favor Anticoagulation Over Structured Surveillance for Patients With Subsegmental Pulmonary Embolism Translated Into Explicit Surveillance Exclusion Criteria

**Clinical conditions that favor anticoagulation over surveillance in 2016 CHEST guideline and expert panel report**[18] [a]	**Explicit criteria excluding patients from structured surveillance, with definitions as indicated**
Proximal DVT detected on bilateral lower-extremity compression ultrasonography (or upper-extremity in patients with indwelling venous catheter)	**Concurrent DVT in upper extremity or proximal lower extremity**<br><br>Those without compression ultrasonography, either at the time of PE diagnosis or in the prior 48h, were categorized as negative for DVT.[b]
Risk factors for recurrent or progressive VTE (if patients are not anticoagulated)	
“Patients who are hospitalized or have reduced mobility for another reason…”	**Inpatient diagnosis of PE**.<br><br>We included only outpatients, whose PE diagnosis was made in the outpatient clinic or ED.<br><br>Chronic immobility, e.g., paraplegia.
“…have active cancer (particularly if metastatic or being treated with chemotherapy);”	**Active cancer.**<br><br>Any cancer treatment (chemotherapy, radiation, surgery) within the prior 6 months, Stage IV disease, or receiving comfort care.
“…or have no reversible risk factor for VTE such as recent surgery”	**No documented reversible VTE risk factor.** These include recent (<3m):<br><br>• Surgery<br>• Hospitalization<br>• Immobilization<br>• Leg trauma<br>• Oral estrogen
Other “risk factors for recurrent VTE”	**Non-reversible risk factor for recurrent VTE**:

© 2023 Rouleau SG et al. *JAMA Network Open*.

eTable 1. Conditions That Favor Anticoagulation Over Structured Surveillance for Patients With Subsegmental Pulmonary Embolism Translated Into Explicit Surveillance Exclusion Criteria

Clinical conditions that favor anticoagulation over surveillance in 2016 CHEST guideline and expert panel report[18 a]	Explicit criteria excluding patients from structured surveillance, with definitions as indicated
	• A history of prior VTE (provoked or not)<br>• Phospholipid antibody disease<br>• Homozygous Factor V Leiden mutation or other known genetic thrombophilic condition<br>• Inflammatory bowel disease
"A low cardiopulmonary reserve"	**Chronic cardiopulmonary disease**:<br>• Chronic pulmonary disease, including asthma<br>• Heart failure, systolic or diastolic
"Marked symptoms that cannot be attributed to another condition"	**Marked symptoms**:<br>• Those with syncope or presyncope[35]<br>• Those whose general appearance was documented as "in distress"
**Clinical conditions not included in 2016 CHEST guideline and expert panel report**	
	**Clinical instability**[c]<br>Defined by one or more of the following documented during the clinic or ED evaluation:<br>• Hypotension: systolic blood pressure <90 mmHg<br>• Tachycardia: heart rate ≥110 bpm<br>• Hypoxemia: peripheral cutaneous oxygen saturation ≤92%
	**Pregnancy**

© 2023 Rouleau SG et al. *JAMA Network Open*.

eTable 1. Conditions That Favor Anticoagulation Over Structured Surveillance for Patients With Subsegmental Pulmonary Embolism Translated Into Explicit Surveillance Exclusion Criteria

**Clinical conditions that favor anticoagulation over surveillance in 2016 CHEST guideline and expert panel report**[18] [a]	**Explicit criteria excluding patients from structured surveillance, with definitions as indicated**
	**Suggested right ventricular strain**:<br>• On CTPA: right ventricular dilatation<br>• On echocardiography[d]<br>• Elevated B-type natriuretic peptide (≥500 pg/mL)[e]<br>• Elevated troponin[e]
	**Concurrent illness requiring inpatient care**

Abbreviations: CTPA, computed tomography pulmonary angiography; DVT, deep vein thrombosis; ED, emergency department; PE, pulmonary embolism; VTE, venous thromboembolism.

[a] Conversely, a high risk of bleeding is noted in the CHEST guideline as a condition that favors non-anticoagulation. We were unable to assess for this across the study population and acknowledge this as a limitation.

[b] Two-thirds of lower-risk patients with subsegmental PE lacked compression ultrasonography. We counted those without compression ultrasonography as negative for DVT. We based this assumption on the multinational SSPE study, which identified concomitant DVT on serial ultrasonography in only 2% of patients with subsegmental PE and lower-risk attributes (e.g., stable outpatients without cancer).[21]

[c] Patients with these non-low-risk vital sign abnormalities were excluded from the current study.

[d] Echocardiographic evidence of right ventricular strain included right ventricular hypokinesis, dysfunction or reduced function, free wall hypokinesis, or systolic septal flattening.

[e] Patients not tested for troponin or B-type natriuretic peptide were categorized as negative.

© 2023 Rouleau SG et al. *JAMA Network Open*.

eTable 2. Excluding Patients With Subsegmental Pulmonary Embolism From Structured Surveillance Without Anticoagulation: Comparing Our Modified CHEST Criteria With Criteria of 2 Ongoing Trials

	Source		
**Surveillance Exclusion Criteria**	**Modified CHEST Criteria used in Current Study (2016)[18]**	**Clinical Surveillance vs. Anticoagulation for Low-risk Patients With Isolated Subsegmental Pulmonary Embolism (SAFE-SSPE)[47 a]**	**STOPping Anticoagulation for Isolated or Incidental Subsegmental Pulmonary Embolism (STOPAPE)[48 a]**
**Proximal Lower-extremity DVT, requires Compression Ultrasonography of Proximal Lower Extremities (and other locations, if indicated)**	Includes “other high-risk locations, such as in upper extremities with central venous catheters”	Includes “upper extremity DVT (subclavian vein or above)”	Includes “thrombus of an unusual site (e.g., upper limbs, associated with a line)”
**Major risk factors for VTE progression or recurrence** (if not anticoagulated)			
Hospitalized or reduced mobility	✓	Hospital-acquired PE	
Active cancer	✓	✓	✓
No documented reversible VTE risk factor. These include recent (<3m):			
Surgery	✓		
Hospitalization	✓		
Immobilization	✓		
Leg trauma	✓		
Oral estrogen	✓		
Non-reversible risk factor for recurrent VTE:			

© 2023 Rouleau SG et al. *JAMA Network Open*.

eTable 2. Excluding Patients With Subsegmental Pulmonary Embolism From Structured Surveillance Without Anticoagulation: Comparing Our Modified CHEST Criteria With Criteria of 2 Ongoing Trials

	Source		
**Surveillance Exclusion Criteria**	**Modified CHEST Criteria used in Current Study (2016)[18]**	**Clinical Surveillance vs. Anticoagulation for Low-risk Patients With Isolated Subsegmental Pulmonary Embolism (SAFE-SSPE)[47 a]**	**STOPping Anticoagulation for Isolated or Incidental Subsegmental Pulmonary Embolism (STOPAPE)[48 a]**
A history of prior VTE	Provoked or not	Unprovoked	Unprovoked
Phospholipid antibody disease	✓		✓
Homozygous Factor V Leiden mutation or other known genetic thrombophilic condition	✓		✓
**Chronic cardiopulmonary disease** (as indicator of impaired cardiopulmonary reserve):			
Chronic pulmonary disease, including asthma	✓		
Heart failure, systolic or diastolic	✓		
**Marked symptoms that cannot be attributed to another condition**			
Syncope or presyncope[35]	✓		
In distress	✓		
**Clinical instability**	• Systolic blood pressure <90 mmHg, or<br>• Heart rate ≥110 bpm, or<br>• O2 saturation <90%	• Systolic blood pressure <100 mm Hg or<br>• O2 saturation <92% at ambient air	• Systolic blood pressure <100 mmHg, or<br>• Heart rate ≥110 bpm, or<br>• O2 saturation <90%

© 2023 Rouleau SG et al. *JAMA Network Open*.

eTable 2. Excluding Patients With Subsegmental Pulmonary Embolism From Structured Surveillance Without Anticoagulation: Comparing Our Modified CHEST Criteria With Criteria of 2 Ongoing Trials

	Source		
**Surveillance Exclusion Criteria**	**Modified CHEST Criteria used in Current Study (2016)[18]**	**Clinical Surveillance vs. Anticoagulation for Low-risk Patients With Isolated Subsegmental Pulmonary Embolism (SAFE-SSPE)[47 a]**	**STOPping Anticoagulation for Isolated or Incidental Subsegmental Pulmonary Embolism (STOPAPE)[48 a]**
**Pregnancy[b]**	✓	✓	✓
**Suggested right ventricular strain**			
On CTPA: right ventricular dilatation	✓		
On echocardiography	✓		
Elevated B-type natriuretic peptide (≥500 pg/mL)	✓		
Elevated troponin	✓		
**Concurrent illness requiring inpatient care**	✓		✓
**Enhancements to CHEST criteria[c]**			
Age <65 years	✓		
Only a single subsegmental PE (not multiple)	✓		
**Other factors**			
Need for therapeutic anticoagulation for another reason[d]	✓	✓	✓

© 2023 Rouleau SG et al. *JAMA Network Open*.

eTable 2. Excluding Patients With Subsegmental Pulmonary Embolism From Structured Surveillance Without Anticoagulation: Comparing Our Modified CHEST Criteria With Criteria of 2 Ongoing Trials

	Source		
**Surveillance Exclusion Criteria**	**Modified CHEST Criteria used in Current Study (2016)[18]**	**Clinical Surveillance vs. Anticoagulation for Low-risk Patients With Isolated Subsegmental Pulmonary Embolism (SAFE-SSPE)[47 a]**	**STOPping Anticoagulation for Isolated or Incidental Subsegmental Pulmonary Embolism (STOPAPE)[48 a]**
Severe renal failure		Creatinine clearance <30ml/min	Stage 5 chronic kidney disease
Severe liver insufficiency		Child-Pugh B or C	
<28 days since first symptoms of proven or clinically suspected Coronavirus disease-19			✓

DVT, deep vein thrombosis; PE, pulmonary embolism; VTE, venous thromboembolism.

[a] We include in the table select criteria most relevant to deliberation on withholding anticoagulation. Complete lists of exclusion criteria from each ongoing trial are available at clinicaltrials.gov.

[b] Pregnant patients were excluded from the 2 ongoing trials also because of contraindications to direct oral anticoagulation used in their treatment arms.

[c] Enhanced criteria were adapted from the SSPE Study.[21]

[d] We addressed this population by excluding patients from the study who were already taking anticoagulation for any reason.

© 2023 Rouleau SG et al. *JAMA Network Open*.

eMethods 2. Definitions of 90-Day Outcomes

Ninety-day outcomes were defined a priori and included major hemorrhage, recurrent VTE, and all-cause mortality, as in our prior PE research. Major hemorrhage was defined by the International Society on Thrombosis and Haemostasis as bleeding at high-risk anatomic locations (intracranial, intraspinal, intraocular, retroperitoneal, intraarticular, pericardial, or intramuscular with compartment syndrome), or overt bleeding with either a reduction of hemoglobin level greater than or equal to 2 g/dL or a transfusion of 2 or more units of red blood cells.[51] Recurrent VTE was defined as a new or expanded abnormality on imaging. Deaths were identified with a health system mortality database that links to the Social Security death master file and the California State Department of Vital Statistics to identify deaths both within and outside of the health care delivery system. We also identified out-of-system medical encounters, using a comprehensive claims database to improve capture of all 90-day outcomes.

© 2023 Rouleau SG et al. *JAMA Network Open*.

eTable 3. Initial Anticoagulation Treatment for Patients With Lower-risk Acute Subsegmental Pulmonary Embolism

Treatment	Lower-risk SSPE (n=229)
	N (%)
**Anticoagulants**	223 (97.4)
Rivaroxaban	112 (50.2)[a]
Dabigatran following 5-10d of low molecular weight heparin	60 (26.9)
Warfarin with a bridge of low molecular weight heparin	31 (13.9)
Low molecular weight heparin alone	18 (8.1)
Apixaban	2 (0.9)
**No anticoagulants**	6 (2.6)

[a] Percentage calculated among patients treated with anticoagulants

[b] See eTable 4 below for a description of these 6 case patients.

© 2023 Rouleau SG et al. *JAMA Network Open*.

eTable 4. Case Patients With Subsegmental Pulmonary Embolism Initially Treated Without Anticoagulation

Case No<br><br>Age in Yrs, Sex	Medical History	History of Present Illness, Physical Examination Findings, Vital Signs, Work-Up, and Meeting Study Criteria for Structured Surveillance	Decision-Making and Clinical Course
Case 1<br><br>47, F	Hypertension, iron deficiency, menorrhagia	Presented to PCP with several days of acute onset, intermittent dyspnea, after recent minor leg trauma. Denied chest pain, coughing, wheezing, hemoptysis. PCP initiated work-up with D-dimer. After CT scan showed multiple subsegmental PE, patient referred to the ED from radiology. No DVT symptoms or signs on examination; in no acute distress with normal mental status. T 36.8°C, SBP 117, HR 87, RR 20, oxygen saturation 99%.[a] Bilateral lower extremity compression US negative for DVT.<br><br>Patient 1[15] met modified CHEST but not enhanced criteria for structured surveillance.	ED physician gave one dose of low-molecular weight heparin and consulted hospital medicine. Hospitalist discussed with patient the diagnosis and the benefits and risks of anticoagulation. The patient agreed with the hospitalist's recommendation to forgo anticoagulation and to wait a couple of weeks for a repeat CTPA. She was discharged home and advised to follow-up with her PCP. The management plan was not communicated to the PCP, whom she saw 6 days later. Her PCP discussed the case with a pulmonologist who recommended 3 months of anticoagulation. Low-molecular weight heparin and dabigatran were ordered by the PCP, and the patient completed a 90-day course of dabigatran. No bleeding complications or VTE recurrence within 90 days.
Case 2<br><br>84, F	Chronic lung disease (asthma), hypertension, paroxysmal SVT, polymyalgia rheumatica	Video visit with PCP for dyspnea with exertion and decreased exercise tolerance for several weeks. No chest pain. No reversible risk factors for VTE identified. On prednisone for polymyalgia rheumatica; prednisone dose increased without improvement in symptoms. Seen in clinic two days later. Dyspnea work-up initiated. D-dimer positive, and PCP referred patient to ED. In ED, no DVT symptoms or signs on examination, in no acute distress, normal mental status. T 36.8°C, SBP 101, HR 99, RR 25, oxygen saturation 94%. CTPA with single subsegmental PE.<br><br>Patient 2 did not meet modified CHEST or enhanced criteria for structured surveillance.	ED physician discussed case with hospitalist who recommended pulmonology consultation. Per pulmonologist, if no DVT on bilateral lower-extremity compression ultrasound, then defer anticoagulation. Patient transferred to observation unit. US negative. Patient remained stable and was discharged home without anticoagulation. Follow-up one day after ED discharge with PCP. PCP discussed case with a different pulmonologist who recommended anticoagulation. As the patient was still symptomatic, PCP initiated rivaroxaban and scheduled follow-up with pulmonology who recommended lifelong anticoagulation. No bleeding complications or VTE recurrence within 90 days.

© 2023 Rouleau SG et al. *JAMA Network Open*.

eTable 4. Case Patients With Subsegmental Pulmonary Embolism Initially Treated Without Anticoagulation

Case No<br><br>Age in Yrs, Sex	Medical History	History of Present Illness, Physical Examination Findings, Vital Signs, Work-Up, and Meeting Study Criteria for Structured Surveillance	Decision-Making and Clinical Course
Case 3<br><br>75, M	Hypertension, systolic heart failure (EF 45%), CAD, VTach with AICD	Presented to ED with worsening dyspnea on exertion for greater than one month and episodes of waking up and feeling short of breath. No chest pain, no wheezing. No reversible risk factors for VTE identified. No DVT symptoms or signs on examination, in no acute distress, normal mental status. T 36.6°C, SBP 168, HR 85, RR 20, oxygen saturation 95%. D-dimer positive. CTPA with motion degradation and bilateral subsegmental PE. BNP elevated at 125.<br><br>Patient 3 did not meet modified CHEST or enhanced criteria for structured surveillance.	ED physician treated symptoms with 20 mg of IV furosemide, which improved the patient's symptoms. No compression US completed while patient in ED. Patient discharged home with plan for outpatient echocardiogram. No anticoagulation initiated at this time, and no VTE surveillance imaging scheduled. Follow-up with PCP via phone call within 48 hours from discharge and was initiated on rivaroxaban for PE. On anticoagulation for 3 months. No major bleeding events or VTE recurrence within 90 days.
Case 4<br><br>61, F	Hypertension, obstructive sleep apnea, sickle cell trait, hyperlipidemia	Presented to the ED with chest pain for one week. She was started on antibiotics for pneumonia by her PCP 6 days prior to ED presentation. No dyspnea, no wheezing. No reversible risk factors for VTE identified. No DVT symptoms or signs on examination, in no acute distress with normal mental status. T 37.2°C, SBP 124, HR 89, RR 22, oxygen saturation 99%. D-dimer elevated. CTPA with single subsegmental PE and resolving lingular pneumonia. Bilateral lower extremity compression US negative for DVT.<br><br>Patient 4 did not meet modified CHEST or enhanced criteria for structured surveillance.	ED physician discussed case with pulmonologist. Pulmonologist discussed with patient that they would recommend anticoagulation, but also possible to not treat given that clot is small and likely incidental finding. Patient deferred starting anticoagulation. Discharged home from ED. Followed-up with PCP and pulmonology within one week of ED discharge. Anticoagulation was not prescribed. No VTE surveillance imaging completed. No major bleeding events or recurrent VTE within 90 days.
Case 5<br><br>77, F	Prior PE, hypertension, paroxysmal atrial fibrillation (not on anticoagulation), chronic lung	Presented to the ED with left sided chest pain for 3 days. No dyspnea, non-exertional. No wheezing. No reversible risk factors for VTE identified. No DVT symptoms or signs on exam, in no acute distress, normal mental status. T 36.8°C, SBP 137, HR 81, RR 23, oxygen saturation 94%. D-dimer elevated. CTPA	ED physician discussed case with hospitalist. Recommended anticoagulation given subsegmental PE, history of prior PE. Patient decided to defer anticoagulation and was discharged from the ED. Follow-up telephone appointment with PCP one day after ED discharge. Patient still declined anticoagulation. PCP ordered repeat CTPA in

© 2023 Rouleau SG et al. *JAMA Network Open*.

eTable 4. Case Patients With Subsegmental Pulmonary Embolism Initially Treated Without Anticoagulation

Case No<br><br>Age in Yrs, Sex	Medical History	History of Present Illness, Physical Examination Findings, Vital Signs, Work-Up, and Meeting Study Criteria for Structured Surveillance	Decision-Making and Clinical Course
	disease (emphysema)	with single subsegmental PE. Bilateral lower extremity compression US was not performed.<br><br>Patient 5 did not meet modified CHEST or enhanced criteria for structured surveillance.	two weeks for surveillance. CTPA was never completed. No ED visits, hospitalizations, or recurrent VTE within 90 days.
Case 6<br><br>65, F	Hypertension	Presented to the ED with two days of dyspnea. Underwent major surgery 10 days prior to presentation. No chest pain, no cough, no wheezing. No DVT symptoms or signs on examination, in no acute distress, normal mental status. T 36.7°C, SBP 150, HR 69, RR 19, oxygen saturation 99%. D-dimer positive. CTPA with single subsegmental PE. Bilateral lower extremity compression US negative for DVT.<br><br>Patient 6 met modified CHEST but not enhanced criteria for structured surveillance.	ED physician discussed case with hospitalist. Patient admitted for low-molecular weight heparin and observation. Repeat d-dimer negative. Hospitalist discussed case with a pulmonologist who did not recommend anticoagulation. Followed-up with PCP and pulmonology within two days. Surveillance DVT US negative 9 days after PE diagnosis. No recurrent VTE.

AICD: automated implantable cardioverter defibrillation; CAD: coronary artery disease; CTPA: computed tomography pulmonary angiogram; DVT: deep vein thrombosis; ED: emergency department; EF: ejection fraction; HR: heart rate; PCP: primary care physician; RR: respiratory rate; SBP: systolic blood pressure; SVT; supraventricular tachycardia: T: temperature; US: ultrasound; VTach: ventricular tachycardia; VTE: venous thromboembolism

[a]We reported the most abnormal vital signs for the encounter, including lowest temperature, lowest blood pressure, highest heart rate, highest respiratory rate, and lowest oxygen saturation.

© 2023 Rouleau SG et al. *JAMA Network Open*.

eTable 5. Exclusion Criteria for Structured Surveillance Without Anticoagulation for Patients With Lower-risk Acute Subsegmental Pulmonary Embolism and Revision of Surveillance Eligibility if Criterion Removed

**Explicit criteria[a] excluding lower-risk patients from structured surveillance**	**Cases excluded[b]** n (% of 229)	**Cases meeting only that row's exclusion criterion[c]**	**Revised Number of Surveillance Eligibility if Row Criterion Removed from Exclusions** **n (% of 666)[d]**
Active cancer	22 (9.6)	4	N/A
No documented reversible VTE risk factor	145 (63.3)[c]	53	88 (13.2)
Non-reversible risk factor for recurrent VTE	42 (18.3)	5	40 (6.0)
Chronic cardiopulmonary disease	68 (29.7)	17	52 (7.8)
Marked symptoms: syncope or presyncope; or "in distress" on examination	15 (6.6)	5	40 (6.0)
Pregnancy	3 (1.3)	2	N/A
Suggested right ventricular strain	30 (13.1)	3	38 (5.7)

Abbreviations: VTE, venous thromboembolism.

[a] See Table 1 for definitions. We follow here that sequence.

[b] Rows are not mutually exclusive. Many patients had more than 1 criterion. The total number excluded was 194.

[c] Numbers in this column are mutually exclusive

[d] Baseline number of surveillance-eligible: 35 (5.3%) of 666-patient cohort. If "no documented reversible VTE risk factor," for example, were removed as a surveillance exclusion, then the number of eligible cases would rise by 53, from 35 to 88.

© 2023 Rouleau SG et al. *JAMA Network Open*.

eReferences

50. Kene M, Arasu V, Warton M, et al. Accurately identifying pulmonary embolism in imaging reports using natural language processing. *Acad Emerg Med*. 2020;27(S1):S76-S77.

51. Schulman S, Kearon C, Subcommittee on Control of Anticoagulation of the S, Standardization Committee of the International Society on T, Haemostasis. Definition of major bleeding in clinical investigations of antihemostatic medicinal products in non-surgical patients. *J Thromb Haemost*. 2005;3(4):692-4. doi:10.1111/j.1538-7836.2005.01204.x

© 2023 Rouleau SG et al. *JAMA Network Open*.
