# Supplementary material for: Prevalence of and Eligibility for Surveillance Without Anticoagulation Among Adults With Lower-Risk Acute Subsegmental Pulmonary Embolism
Source: JAMA Netw Open. 2023 Aug 2;6(8):e2326898. doi: 10.1001/jamanetworkopen.2023.26898 (PMC10398409; doi:10.1001/jamanetworkopen.2023.26898)
Supplement: Supplement 2. — Data Sharing Statement [file jamanetwopen-e2326898-s002.pdf]

Data Sharing Statement

Rouleau. Prevalence of and Eligibility for Surveillance Without Anticoagulation Among Adults With Lower-Risk Acute Subsegmental Pulmonary Embolism. *JAMA Netw Open*. Published August 02, 2023. doi:10.1001/jamanetworkopen.2023.26898

Data
Data available: No
